# Physiological dynamics as indicators of plant response to manganese binary effect

**DOI:** 10.3389/fpls.2023.1145427

**Published:** 2023-04-12

**Authors:** Xu Zhenggang, Fan Li, Zheng Mengxi, Zhao Yunlin, Huang Huimin, Yang Guiyan

**Affiliations:** ^1^ College of Forestry, Northwest A&F University, Yangling, China; ^2^ Hunan Research Center of Engineering Technology for Utilization of Environmental and Resources Plant, Central South University of Forestry and Technology, Changsha, Hunan, China; ^3^ Department of Environmental Monitoring, Changsha Environmental Protection College, Changsha Hunan, China

**Keywords:** *Broussonetia papyrifera*, physiological response, antioxidant enzyme, long-term stress, heavy metal

## Abstract

**Introduction:**

Heavy metals negatively affect plant physiology. However, plants can reduce their toxicity through physiological responses. *Broussonetia papyrifera* is a suitable candidate tree for carrying out the phytoremediation of manganese (Mn)-contaminated soil.

**Methods:**

Considering that Mn stress typically exerts a binary effect on plants, to reveal the dynamic characteristics of the physiological indexes of *B. papyrifera* to Mn stress, we conducted pot experiments with six different Mn concentrations (0, 0.25, 0.5, 1, 2, and 5 mmol/L) for 60 days. In addition to the chlorophyll content, malondialdehyde (MDA), proline (PRO), soluble sugar, superoxide dismutase (SOD), catalase (CAT), peroxidase (POD), the absorption and transfer characteristics of Mn, and root structure were also measured.

**Results:**

Phytoremedial potential parameters such as the bioconcentration factor (BCF) and translocation factor (TF) displayed an increasing trend with the increase of Mn concentration. At lower Mn concentrations (<0.5 mmol/L), the TF value was **<**1 but crossed 1 when the Mn concentration exceeded 100 mmol/L. The Mn distribution in various tissues was in the following order: leaf > stem > root. The root structure analysis revealed that low-level concentrations of Mn (1 mmol/L) promoted root development. Mn concentration and stress duration had significant effects on all measured physiological indexes, and except soluble sugar, Mn concentration and stress time displayed a significant interaction on the physiological indexes.

**Discussion:**

Our study demonstrates that the physiological indexes of *B. papyrifera* display dynamic characteristics under Mn stress. Thus, during the monitoring process of Mn stress, it appears to be necessary to appropriately select sampling parts according to Mn concentration.

## 1 Introduction

Recently, heavy metal pollution in soil has become increasingly serious due to ore smelting and the excessive use of chemical fertilizers (Ali et al., 2013b). Pollution poses challenges to plant growth, agricultural production, and ecosystem health and even threatens human health and food safety (Alsherif et al., 2022; Sarker et al., 2022; Wen et al., 2022). Heavy metals in soil persist for a long time because of their poor mobility and resistance to biodegradation. Under heavy metal stress, plants undergo changes in molecules, physiology, and morphology (Xu et al., 2018; Zhang et al., 2020). Physiological responses such as those involving malondialdehyde (MDA), superoxide dismutase (SOD), peroxidase (POD), and proline (PRO) may alleviate the toxicity of heavy metals by regulating osmosis or scavenging reactive oxygen species (ROS) (Ali et al., 2014; Panda et al., 2016). Physiological indexes have been determined by almost all plant studies that have researched heavy metal stress (Ali et al., 2013a; Ali et al., 2013b; Ali et al., 2014). Plant physiological responses are affected by heavy metal concentration and stress time, and thus, it is critical to select the appropriate concentration and stress time to accurately reflect their relationship (Ghori et al., 2019; Gong et al., 2021). Until now, most experimental designs have focused on examining the physiological response of plants under different heavy metal concentrations (Tauqeer et al., 2016); however, these experiments have been conducted for limited time periods. Under environmental stress, plants adopt a stress-adaptation reaction process, and consequently, an inappropriate time window of experimental observation may lead to incorrect conclusions about their physiological responses. Therefore, it is necessary to explore the time-series characteristics of physiological indexes of plants under different heavy metal concentrations.

Among the heavy metals, cadmium (Cd) and lead (Pb) do not possess any beneficial role in plants but become toxic at higher concentrations. However, other metals such as zinc (Zn), copper (Cu), and nickel (Ni) are essential micronutrients, as they are required in trace amounts and work as cofactors for various enzymes (Ghori et al., 2019). Manganese (Mn), a trace element that is used in plant metabolism, prevents oxidative damage and plays a key role in the development and growth of plants (Li et al., 2019). Excessive Mn soil accumulation pollutes the surrounding environment and affects plant growth (Xu et al., 2019a; Nong et al., 2023). Furthermore, Mn stress produces toxic effects in plants, and high concentrations or long-term Mn accumulation may lead to plant death (Pan et al., 2018). To reduce the environmental harm of Mn, phytoremediation technology has been widely promoted, which possesses the advantages of being environmental-friendly, cost-effective, and not producing secondary pollution (Huang et al., 2022).

Paper mulberry (*Broussonetia papyrifera*) is a deciduous tree that belongs to the Moriaceae family (Wan et al., 2022). It is characterized by rapid growth, strong adaptability, wide distribution, and easy reproduction (Zhou et al., 2021). It can survive in mining areas with serious heavy metal pollution (Zhenggang et al., 2022). In recent years, multiple studies have reported that *B. papyrifera* can be used as a pioneer species for phytoremediation in mining areas, which effectively improves the environmental quality of polluted soils (Huang et al., 2019; Huang et al., 2020; Nong et al., 2023). This tree possesses a strong enrichment ability and transfers various heavy metals, and its comprehensive bioconcentration index can reach a value of 2.93 (Zhao et al., 2014). Until now, only a few studies have examined the physiological and biochemical responses of *B. papyrifera* to heavy metal stress, and a majority of studies have primarily focused on drought, salt, and alkali stress (Li et al., 2011). In recent years, although the physiological response of *B. papyrifera* to Cd and Mn stress has been studied, changes in physiological indexes over longer time periods have not been studied (Huang et al., 2019; Zhang et al., 2020).

Monitoring plants’ physiological processes during long-term heavy metal stress is central to gaining insights into their physiological coping strategies in response to environmental stress. The temporal changes of physiological indexes can be used to monitor plant survival status. For example, chlorophyll a fluorescence has been used to monitor the physiological status of plants under abiotic stress conditions (Kalaji et al., 2016). While heavy metals are known to accumulate in plants over time, whether the physiological responses of plants to heavy metals are consistent with the extension of stress time remains poorly understood. We hypothesized that the time-series characteristics of plant physiological indexes under Mn stress are different. In this study, we determined the physiological response of *B. papyrifera* under long-term Mn stress (60 days) by carrying out several experiments described as follows: i) the impact of varying Mn concentrations on the growth of and accumulation in *B. papyrifera*, ii) the effect of Mn stress on chlorophyll synthesis in *B. papyrifera*, and iii) the effect of different Mn concentrations on the physiological responses of *B.papyrifera*, including antioxidant enzyme activity, soluble sugar, and PRO and MDA activity, at different time points. This study provides a framework for determining the physiological indexes of *B. papyrifera* at the appropriate time under different Mn concentrations.

## 2 Materials and methods

### 2.1 Experimental materials and Mn stress treatments

One-year-old seedlings with good growth and developed root systems were purchased from Anhui Zhongke Anyue Forestry Science and Technology Development Co., Ltd. (http://www.ahzkayly.com/display/264987.html) and were used for the pot experiments. Fertile soil, which mainly consisted of peat, vermiculite, slag, and perlite, was purchased from Lianyungang Hengaoda Fertilizer Technology Co., Ltd. (http://www.ampcn.com).

Healthy seedlings were transplanted into flowerpots with nutrient-enriched soil for the Mn stress experiment. During the experiment, six different concentrations of Mn solution (0, 0.25, 0.5, 1, 2, 5 mmol/L) were prepared, and then the same amount of solution (100 ml) was used to irrigate each experimental group. To prevent Mn loss from the flowerpots, a tray was placed under each flowerpot. The pots were kept in a greenhouse at 26–30°C with a 14:10-h light:dark cycle (Huang et al., 2019). The bottom tray was watered every 5 days until harvest, which was sufficient to meet the water demands of the plants.

### 2.2 Determination of the root structure

After 60 days of cultivation at different Mn concentrations, the roots of the plants were washed with deionized water and dried with filter paper. An Epson scanner (11000XL, Epson scanner, Japan) was used to scan and obtain images of the root system of the plants. The total root length, surface area, number of root tips, and number of crosses of the plant root system were analyzed using the WinRhizo Pro software (2013e, Regent Instruments Inc., Canada) according to the instruction manual (Huang et al., 2019).

### 2.3 Determination of physiological indexes

After Mn treatment, the physiological indexes of the *B. papyrifera* leaves were measured at different time points (12 h, 15 days, 30 days, 45 days, and 60 days) for all the experimental groups. The chlorophyll content was determined using the colorimetric method (Zhang et al., 2020), and the MDA content was determined using the thiobarbituric acid (TBA) method (Ali et al., 2014). The PRO content and soluble sugar content were determined using the colorimetric method and the anthrone colorimetric method, respectively (Huang et al., 2019). Furthermore, antioxidant enzyme activities were analyzed and SOD activity was determined using the hydroxylamine method (Ali et al., 2014). Then, the POD activity was assayed using the colorimetric method, and the CAT activity was determined using the ammonium molybdate colorimetric method (Ali et al., 2013b; Ali et al., 2014). The above indexes were tested using kits provided by the Nanjing Jiancheng Bioengineering Institute (http://www.njjcbio.com/), and the specific procedures were performed according to the manufacturer’s instructions.

### 2.4 Determination of Mn content in various tissues of *Broussonetia papyrifera* and soil

The Mn content in various tissues of *B. papyrifera* and soil was determined according to the methods used in previous reports (Huang et al., 2019; Huang et al., 2020). After harvesting, the fresh leaves, stems, and roots were dried in an oven at 105°C for 30 min and 70°C for 72 h and then ground into powders. Tissue samples (0.2 g) were weighed in a 100-ml Erlenmeyer flask inside a fume hood (SFS-FH-001 fume hood, China). Next, 10 ml of nitric acid was added, and the mixture was shaken well, covered with a curved neck funnel, and allowed to stand overnight. The next day, the samples were heated on an electric heating plate (RJM-10 electric heating plate, China) at 160°C, 170°C, 180°C, and 190°C for 1 h each until the brown gas disappeared, and then the samples were removed and cooled. Then, 3 ml of perchloric acid was added while gradually increasing the temperature of the hot plate to 200°C over a period of 1–2 h. When the solution in the Erlenmeyer flask was colorless and transparent, the solution was cooled to a constant volume of 50 ml. After appropriate dilution, an Atomic Emission Spectrometer (7510 Atomic Emission Spectrometer, Japan) was used to determine the Mn concentration (Pan et al., 2018). Mn content in the soil was also determined with air-dried soil samples (0.5 g).

### 2.5 Statistical analysis

SPSS 20.0 software (SPSS Inc., Chicago, IL, USA) and Origin 9 (OriginLab, Northampton, MA, USA) were used to analyze the experimental data and to draw the figures, respectively. The Kolmogorov–Smirnov test was used to examine the normality of the data distribution. One-way analysis of variance (ANOVA) was used to determine whether the difference between the experimental groups was significant (*P* < 0.05) in terms of the root structure and the Mn content in the tissue, while the physiological indexes were analyzed using a two-factor ANOVA. The least significant difference (LSD) test was performed to detect homogeneity of variance (*P* > 0.05). In addition, the translocation factor (TF) was computed as the ratio of Mn content in the aboveground parts to that in the underground parts, and the bioconcentration factor (BCF) was calculated as the ratio of the Mn concentrations in the plant to Mn concentrations in the soil (Huang et al., 2019; Huang et al., 2022). The Mantel test was also employed to explore the relationships between heavy metal adsorption traits, root traits, and physiological indexes. All measurements were repeated three times, and the data were expressed as mean values ± standard deviation (SD).

## 3 Results

### 3.1 Absorption and enrichment of Mn by *Broussonetia papyrifera*

After 60 days of Mn stress treatment, the Mn concentration in each part of *B. papyrifera* changed. The results showed that with the increase of the Mn concentration in soil, the Mn content in the roots, stems, and leaves of *B. papyrifera* also increased (**Table 1**). In all treatment groups, Mn accumulated in different tissues of *B. papyrifera* (in the following order: leaf > stem > root), indicating that the increase of Mn concentration in the environment promoted the transportation of Mn to aerial parts and was mainly concentrated in the leaves. TF and BCF could be used to evaluate the phytoremediation ability of heavy metal pollution in soils (Assunção et al., 2003). In terms of Mn enrichment and transfer characteristics of *B.papyrifera*, the BCF value showed a trend of first increasing and then reaching saturation with increasing soil Mn concentration, and the maximum BCF value was 0.77 under the 2-mmol/L Mn treatment. As the Mn concentration increased, the TF value increased from 0.89 to 1.22.

**Table 1:** The Mn bioaccumulation and translocation by *Broussonetia papyrifera* with different Mn concentrations for 60 days.

Concentration (mmol/L)	Mn in plant tissue (mg/kg, DW)			BCF	TF
	Root	Stem	Leaf		
0	17.25 ± 4.88a	20.25 ± 8.40 a	52.88 ± 22.87b	0.60 ± 0.03b	0.89 ± 0.13b
0.25	32.14 ± 6.18a	35.21 ± 7.29 a	85.69 ± 18.55ab	0.62 ± 0.05b	0.93 ± 0.05b
0.5	42.71 ± 34.13a	65.48 ± 56.84 a	59.37 ± 34.48b	0.66 ± 0.08b	1.05 ± 0.08ab
1	27.95 ± 6.29a	49.64 ± 8.32a	99.29 ± 33.81ab	0.75 ± 0.02ab	1.14 ± 0.09ab
2	40.38 ± 29.55a	69.67 ± 31.00a	115.49 ± 14.09a	0.77 ± 0.02a	1.15 ± 0.05ab
5	39.96 ± 15.91a	64.62 ± 25.48a	128.11 ± 15.52a	0.73 ± 0.04ab	1.22 ± 0.03a

The data are presented as mean ± SD of three replicates. Different letters indicate significant differences between the same indexes (P < 0.05).

### 3.2 Root structural changes of *Broussonetia papyrifera* under Mn stress

After 60 days, different Mn concentrations showed different effects on the root structure of *B. papyrifera* (**Figure S1**). As the Mn concentration increased, the total root length, surface area, root tip number, and crossing number of *B. papyrifera* increased first and then decreased (**Table 2**). Following 1 mmol/L of Mn treatment, all indexes reached their maximum values and were significantly higher than the control (0 mmol/L). In addition, when the Mn concentration was 0.25–5 mmol/L, the total root length, surface area, root tip number, and cross number of *B. papyrifera* were greater than those of the control group (0 mmol/L).

**Table 2:** Changes of root characteristics of *Broussonetia papyrifera* under different Mn concentrations.

Concentration (mmol/L)	Length (cm)	Surface area (cm²)	Crossings	Tips
0	416.77 ± 20.22b	19.90 ± 2.55a	1,255.33 ± 754.29b	2,807.67 ± 35.84b
0.25	429.27 ± 16.52b	22.14 ± 2.68a	1,346.33 ± 213.15b	2,861.67 ± 143.67b
0.5	516.78 ± 92.06ab	22.56 ± 3.89a	1,746.33 ± 185.48ab	4,084.67 ± 925.90ab
1	587.16 ± 93.41a	25.29 ± 3.68a	1,990.33 ± 211.13a	5,544.00 ± 1,083.33a
2	527.99 ± 78.12ab	25.19 ± 1.04a	1,321.67 ± 89.31ab	4,388.33 ± 2,696.97b
5	455.19 ± 56.02b	24.98 ± 6.77a	1,296.33 ± 218.79ab	4,104.00 ± 1,290.09b

The data are presented as mean ± SD of three replicates. Different letters indicate significant differences between the same indexes (P < 0.05).

### 3.3 Chlorophyll content changes of *Broussonetia papyrifera* under Mn stress

**Figure 1:**
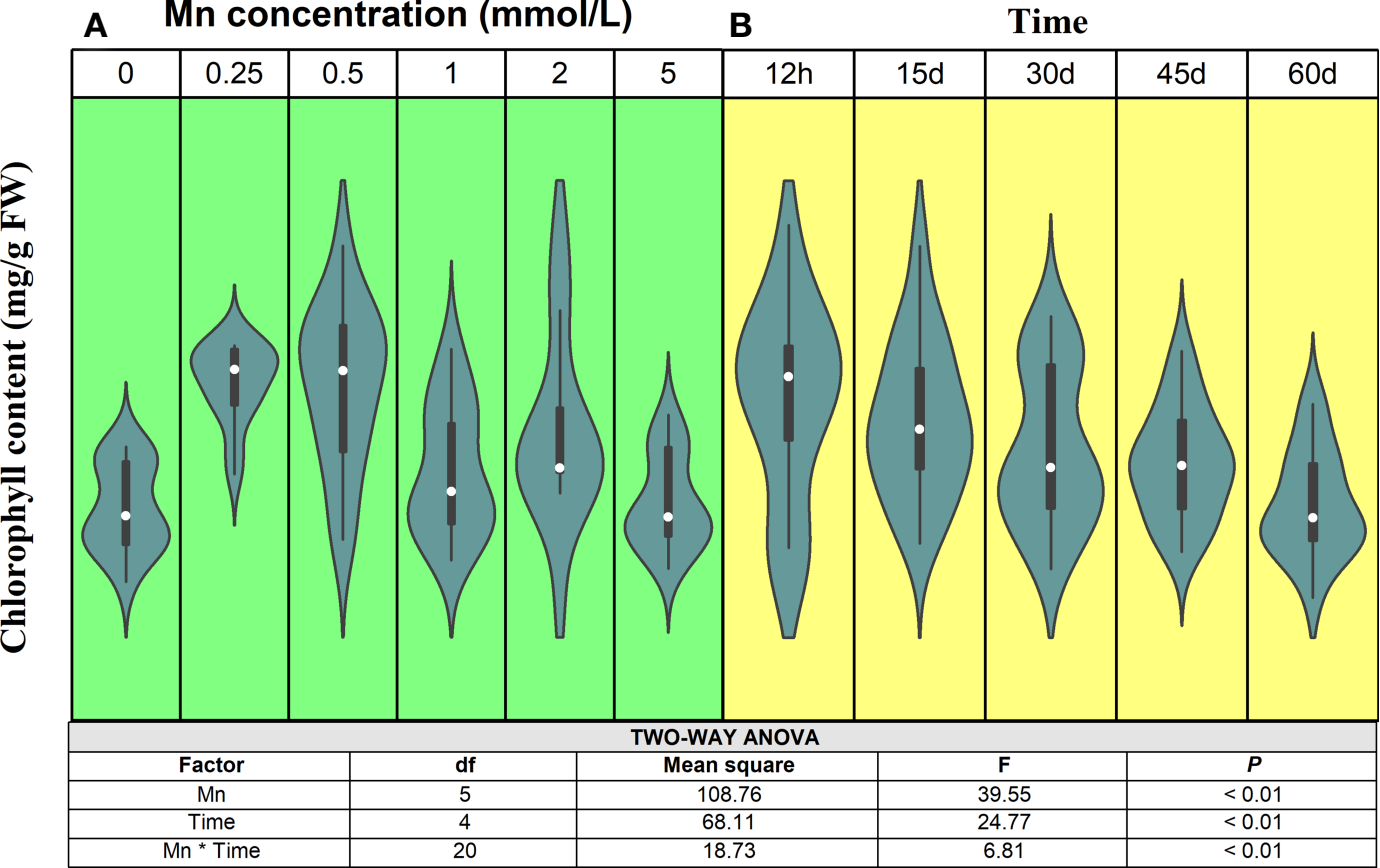
The change trends of chlorophyll content of *Broussonetia papyrifera* with different Mn concentrations **(A)** and time **(B)**. The table showed the results of two-way ANOVA. Mn, Mn concentration effect; Time, time effect; Mn * Time, the interactive effect of Mn concentration and time.

Mn is an essential element for chlorophyll synthesis (Ono et al., 1992), and the effect on chlorophyll content showed typical binary characteristics (**Figure 1**). Statistical analysis showed that both Mn concentration and stress time had significant effects on the chlorophyll content of *B. papyrifera* (*P* < 0.01). Meanwhile, there was also a significant interaction between Mn concentration and stress time (*P* < 0.01). Specifically, as Mn concentration increased, the chlorophyll content of *B. papyrifera* increased first and then decreased. Over short periods (12 h), the chlorophyll content decreased until the Mn concentration reached 5 mmol/L. With longer stress times (15 and 30 days), when the Mn concentration reached 1 mmol/L, the chlorophyll content of *B. papyrifera* began to decrease. When the treatment time reached 45–60 days, the chlorophyll content began to decrease, even when the Mn concentration was very low (0.5 mmol/L). Considering the time scale, the chlorophyll content decreased as the Mn stress was prolonged. In fact, when there was no Mn stress (0 mmol/L), the chlorophyll content increased constantly. At different Mn concentrations, the chlorophyll content of *B. papyrifera* decreased with increasing stress times (**Figure S2**).

### 3.4 Changes in MDA, PRO, and soluble sugar in *Broussonetia papyrifera* under Mn stress

**Figure 2:**
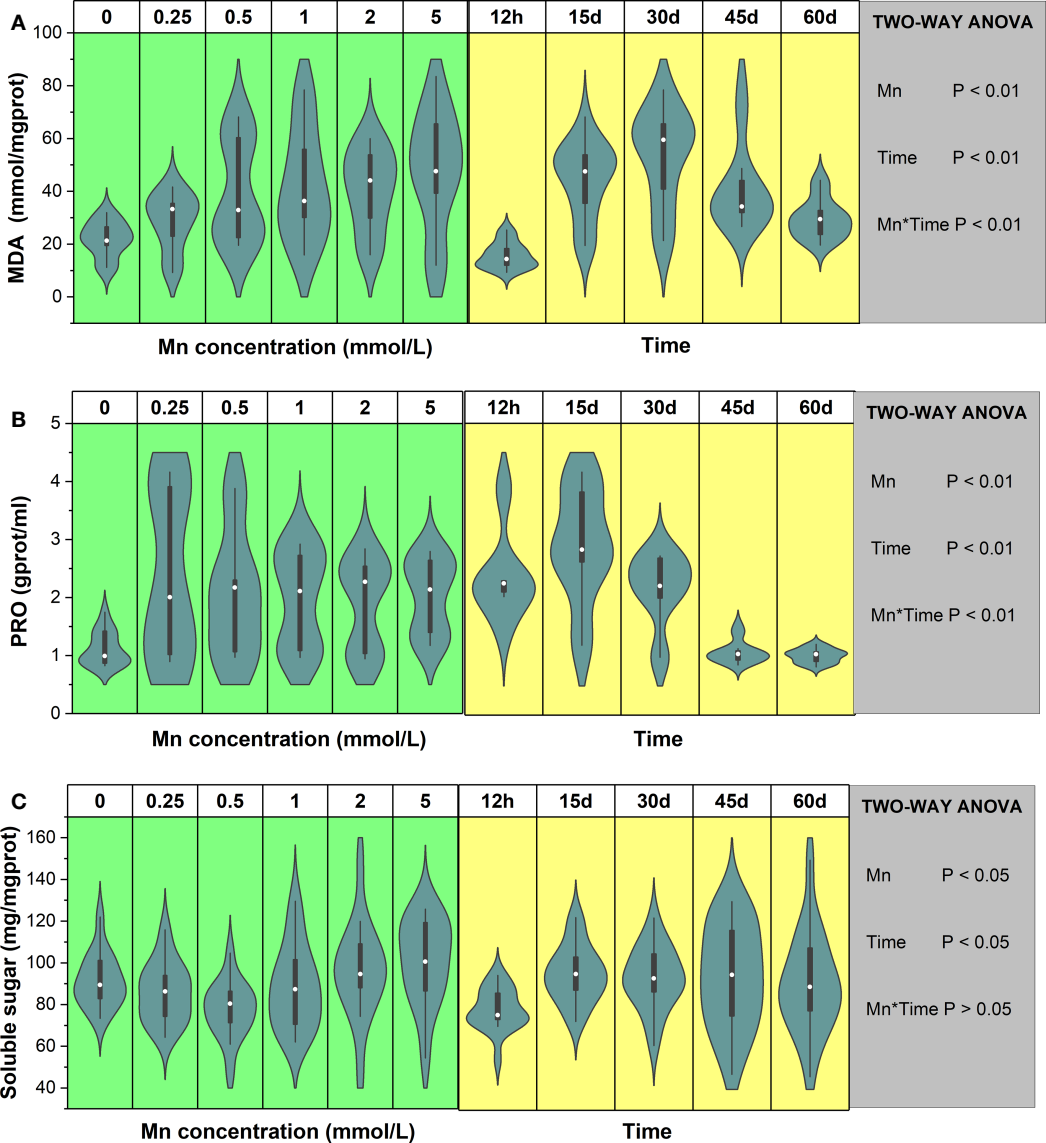
The change trends of MDA **(A)**, proline **(B)**, and soluble sugar **(C)** of *Broussonetia papyrifera* with different Mn concentrations and time. The table showed the results of two-way ANOVA. Mn, Mn concentration effect; Time, time effect; Mn * Time, the interactive effect of Mn concentration and time.

Mn stress had a significant effect on MDA concentration (*P* < 0.01), and MDA content changed significantly over time (**Figure 2A**). With increasing Mn concentration, MDA content also increased. There was a significant interaction between Mn concentration and exposure time (*P* < 0.01), and different Mn concentrations had different characteristics at different times. During the early stage of Mn stress (12 h–30 days), MDA content showed a trend of rising first and then decreasing. When the stress time was 12 h, the maximum value of MDA appeared at the Mn concentration of 0.5 mmol/L. As stress time became extended, the peak of MDA appeared when the concentration of Mn was high. When the stress time of Mn exceeded 45 days and the Mn concentration was 5 mmol/L, MDA content still did not decrease. Overall, under different Mn concentrations, the MDA content showed a consistent trend of first increasing and then decreasing. Compared with the control group, the maximum MDA content of each treatment group increased by 110% (12 h), 204% (15 days), 237% (30 days), 22% (45 days), and 60% (60 days) (**Figure S3A**).

The PRO content results in *B. papyrifera* revealed that Mn stress had a significant effect on PRO content (*P* < 0.01, **Figure 2B**). Overall, compared with the control group (0 mmol/L), Mn stress significantly increased the PRO content of *B. papyrifera.* There was also a significant interaction between Mn concentration and stress time (*P* < 0.01). Specifically, for different stress durations, PRO content showed different trends with increasing Mn concentration (**Figure S3B**). Over short stress periods (12 h–30 days), the PRO content of *B. papyrifera* showed a large range of changes as the Mn concentration increased. At the late stage of Mn stress (45–60 days), as Mn concentration increased, the PRO content did not vary much but remained at a relatively low level. Furthermore, PRO content decreased with time when the Mn concentration was 0 mmol/L. Overall, in the presence of Mn stress, the PRO content increased first and then decreased.

A two-way ANOVA showed that both Mn concentration and time had significant effects on the soluble sugar content of *B. papyrifera* (*P* < 0.05), and the soluble sugar content changed significantly with time (**Figure 2C**). Meanwhile, the interaction between Mn concentration and time had little effect on soluble sugar content (*P* > 0.05). Specifically, with increasing Mn concentration, soluble sugar content decreased first and then increased. In most cases, the lowest value of soluble sugar appeared at the Mn concentration of 0.5 mmol/L (**Figure S3C**). For all Mn concentrations, the soluble sugar content of *B. papyrifera* was higher after long-term stress than that after 12 h of stress. Over short periods of time (12 h–15 days), the soluble sugar content was not significantly different, and the maximum soluble sugar content was observed in the control group (0 mmol/L). After 30, 45, and 60 days of Mn stress, the soluble sugar content reached the maximum value after treatment with the highest concentration of Mn (5 mmol/L).

### 3.5 Changes in antioxidant enzyme activity in *Broussonetia papyrifera* under Mn stress

**Figure 3:**
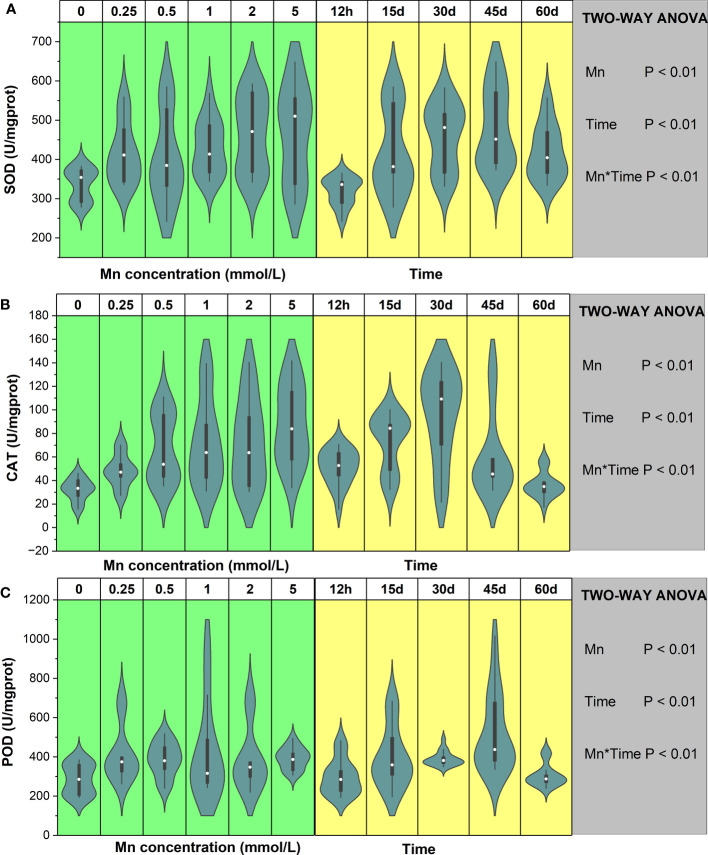
The change trends of SOD **(A)**, CAT **(B)**, and POD **(C)** of *Broussonetia papyrifera* with different Mn concentrations and time. The table showed the results of two-way ANOVA. Mn, Mn concentration effect; Time, time effect; Mn * Time, the interactive effect of Mn concentration and time.

The results of this study demonstrated that the SOD activity of *B. papyrifera* significantly changed under Mn stress and that stress duration significantly affected SOD activity (*P* < 0.01, **Figure 3A**). There was a significant interaction between Mn concentration and time (*P* < 0.01). Compared with the control group, Mn stress significantly increased the SOD content of *B. papyrifera*. Additionally, after long-term stress, the SOD activity of *B. papyrifera* was significantly higher than after 12 h of stress (**Figure S4A**). Over short periods of time (12 h–30 days), the maximum value of SOD appeared at low Mn stress levels (<2 mmol/L). After 45 and 60 days of Mn stress, the SOD content increased with increasing Mn concentration and reached the maximum value at the highest concentration of Mn (5 mmol/L). In contrast, the SOD content showed the same trend in all the groups, increasing first and then decreasing as stress time was prolonged. For low Mn concentrations (0.25 – 0.5 mmol/L), the SOD content reached the maximum value at 15 days. Under the highest Mn concentration (5 mmol/L), the SOD content reached the maximum value at the late stage of Mn stress (45 days). The maximum SOD content of each treatment group increased by 19% (12 h), 100% (15 days), 51% (30 days), 65% (45 days), and 42% (60 days) compared with the control group (**Figure S4A**).

The CAT activity was significantly affected by Mn stress and stress duration (*P* < 0.01), and there was a significant interaction between the above factors (*P* < 0.01, **Figure 3B**). As the Mn concentrations increased, the CAT activity of *B. papyrifera* also increased. During the early Mn stress period (12 h–30 days), the maximum value of CAT appeared at low stress levels (<1 mmol/L) and increased by 115% (12 h), 172% (15 days), and 341% (30 days) compared with the control group. At the late stage of Mn stress (45–60 days), CAT levels reached their maximum with the highest concentration of Mn (5 mmol/L), which increased by 235% (45 days) and 117% (60 days) compared with the control group (**Figure S4B**). Additionally, the CAT content under different Mn concentrations initially increased and then decreased with prolonged stress time. As the Mn concentration increased, the activity of CAT continued to increase, indicating that CAT continued to play a regulatory role under Mn stress.

Although Mn concentration and stress time had significant effects on the POD activity of *B. papyrifera* (*P* < 0.01), the variation was large (**Figure S4C**). Compared with 0 mmol/L of Mn, Mn stress increased POD activity, but the increase was not significant. During the early stage of Mn stress (12 h–45 days), the maximum value of POD appeared at low stress levels (<1 mmol/L). At 60 days, the POD content reached the maximum value at the highest concentration of Mn (5 mmol/L). Overall, the results demonstrated that the POD content under different Mn concentrations showed a trend of increasing first and then decreasing with time (**Figure 3**). For low Mn stress (0.25–0.5 mmol/L), the POD content reached the maximum value at the early stage of stress. Additionally, compared with the control group, the maximum POD content of each treatment group increased by 130% (12 h), 218% (15 days), 24% (30 days), 135% (45 days), and 45% (60 days).

## 4 Discussion

### 4.1 Mn pollution and Broussonetia papyrifera

Heavy metal pollution in soil is a major threat to plant production and ecosystem health (Wen et al., 2022). To tackle this issue, phytoremediation is a widely employed approach, which uses the adsorption characteristics of plants to extract and remove pollutants in soil. Heavy metal tolerance and enrichment characteristics are decisive factors in determining whether a species is suitable for planting in contaminated soil. Studies have shown that Mn toxicity in plants differs from those of other heavy metals, and it was first observed in the leaves (Zeng et al., 2004). Mn directly participates in the redox reaction of the electron transport system during photosynthesis and plays a key role in the stability of the chloroplast membrane structure (Petolino and Collins, 1985). Furthermore, the accumulation of Mn affects photosynthesis, by altering the photosynthetic electron transfer process, and significantly decreases the chlorophyll content (Oliveira et al., 2022).

Compared with herbs, woody plants have an advantage in landscape restoration, as they possess a considerable capacity to absorb various heavy metals (Xu et al., 2019b; Nong et al., 2023). *B. papyrifera* is widely distributed and has a long history of use in papermaking, bark cloth making, medicine, and livestock breeding (Peng et al., 2019). In recent years, *B. papyrifera* has been widely used to resolve heavy metal pollution, especially Cd and Mn pollution (Huang et al., 2019; Peng et al., 2019; Huang et al., 2020; Zhang et al., 2020; Huang et al., 2022). In this study, we noticed that an increase in soil Mn led to increased Mn concentration in all tissues of *B. papyrifera*, indicating that the root system of this plant absorbs Mn from the soil and transports it to various tissues. Our results concerning the adsorption characteristics of *B. papyrifera* to Mn are consistent with those of previous studies, which reported *B. papyrifera* to be an ideal candidate plant for the phytoremediation of Mn soil pollution (Huang et al., 2019). However, our finding that the concentration of Mn in the roots of *B. papyrifera* was significantly lower than that in the leaves is contrary to the distribution of Cd in *B. papyrifera* (Zhang et al., 2020). These results likely reflect the differences in the characteristics of Cd and Mn. It is possible that even a small amount of Cd is toxic to plants; however, a low concentration of Mn, being an essential element, is essential for enzymatic activity and promotes plant growth and survival (Zhang et al., 2010). Furthermore, we found that Mn had a certain degree of influence on the root structure of *B. papyrifera* across different concentrations. We feel that this phenotype may be an adaptive protection mechanism for plants against initial Mn stress. This interpretation is supported by a previous study: the more developed the root structure, the lower the relative concentration of heavy metals in the plant (Zacchini and de Agazio, 2004).

### 4.2 Effect of Mn on physiological indexes of *Broussonetia papyrifera*

In this study, the chlorophyll content first increased and then decreased, indicating that low concentrations of Mn likely promoted chlorophyll content to enhance photosynthesis. However, when the Mn concentration exceeded the tolerance limit of the plant, it likely inhibited the synthesis of the light-harvesting complex in the chloroplast sheet, and the activity of related enzymes was also inhibited. Notably, our results of the effects of Mn stress on the chlorophyll content of *B. papyrifera* differ from those of Cd (Zhang et al., 2020).

MDA is a product of membrane lipid peroxidation, which forms insoluble compounds with active substances such as PRO, nucleic acids, and amino acids, thus interfering with normal cellular activities (Monteiro et al., 2009). Previous studies have shown that under a certain degree of heavy metal stress, various cellular protective mechanisms can maintain normal cellular activity. However, if the stress intensity is too high, it would cause cell metabolism disorders, free radical accumulation, and increased membrane lipid peroxidation and MDA content (Song et al., 2022). In the present study, we found that the MDA content in *B. papyrifera* significantly increased over time under different Mn concentrations. It is likely that Mn stress induced a large amount of ROS in the *B. papyrifera* plant, which damaged the cell membrane structure system and led to increased MDA content. The MDA content then began to decrease, indicating the adaptation by plants to the stress environment (Giannakoula et al., 2021).

We also noticed an increase in PRO levels in response to Mn stress. PRO is an important osmotic adjustment substance for the physiological regulation of plants. It plays an important role in the osmotic adjustment of cells, stabilization of cell structure, and reduction of oxidation (Mehta and Gaur, 1999). Under adverse conditions (drought, salt alkali, and heavy metal pollution), PRO accumulates in the cytoplasm to perform an osmotic adjustment to reduce toxicity (Xu et al., 2018). Therefore, PRO, similar to MDA, can indirectly predict the stress resistance of plants and can be used as a physiological indicator of senescence under various stressors (Huang and Wang, 2010).

Soluble sugar is the main osmotic regulator in plants, which stabilizes cell membranes and protoplast colloids and protects cells from high concentrations of inorganic ions. Under osmotic stress, plants can reduce their osmotic potential by accumulating soluble sugars to adapt to the changes in the external environment. In this study, under the stress of low concentrations of Mn, soluble sugar content increased in *B. papyrifera*. This observation suggests that plants likely adjusted their cell osmotic pressure to reduce the damage. The increase in soluble sugar and other organic small-molecule solutes could reduce the water potential in cells to achieve absorption of water from surrounding cells. The soluble sugar content of *B. papyrifera* decreased with time, which may have been caused by a decrease in carbohydrates and plant respiration.

To remove excessive reactive oxygen free radicals and maintain normal plant growth, antioxidant enzymes in plants were significantly increased. Antioxidant enzymes scavenge active oxygen free radicals in the presence of heavy metals (Xu et al., 2019b). In general, the removal and production of active oxygen free radicals in plants are in a relatively stable state, and excess active oxygen free radicals are mainly eliminated by enzyme systems such as SOD, CAT, and POD. Therefore, the increased activity of these protective enzymes can enhance the ability of plants to scavenge active oxygen free radicals, thereby reducing damage (Mandal et al., 2013). We noticed that the SOD, CAT, and POD contents first increased and then decreased with time under different Mn concentrations. This may be one of the mitigation mechanisms of *B. papyrifera* to Mn stress, which is consistent with other adaptive mechanisms under heavy metal stress (Shi et al., 2010). As the stress time extended, the activities of the three antioxidant enzymes in the high Mn concentration decreased, and the range of changes in CAT content was much greater than that of SOD and POD, which indicated that CAT played a major regulatory role at high concentrations. However, because oxygen free radicals accumulate over time, it is still necessary to regulate and reduce lipid peroxidation by enzyme systems such as SOD, POD, and CAT.

### 4.3 Indicators of the binary effect of Mn

Based on the binary effect, it is necessary to find suitable indicators to distinguish the effect of Mn. Changes in growth and biomass are the first visible responses that plants show under heavy metal stress, especially in plant roots, which are in direct contact with contaminated soil (Assunção et al., 2003). With the aggravation of Mn toxicity, root growth is also disrupted likely caused by cytoplasm damage, rupture in meristematic cells and root cap cell membranes, resulting in the root tip meristem becoming the most severely damaged part in Mn poisoning (Santandrea et al., 1998). Based on the response of plant phenotypes to heavy metal stress, plant phenomics has been paid more attention in recent years. The novel computer visualization for plant phenotyping was recently used to detect the adsorption characteristics to heavy metals, and this tool was found to be more suitable for leguminous species than trees and grasses (Al-Lami et al., 2021). Furthermore, the effect of Cd on the roots of rice was explored by a comprehensive analysis of the root phenotype (Wang et al., 2023). Thus, it appears that although plant phenotype is an important indicator of the impact of heavy metals on plants, its accuracy is often low, and it may even cause fatal damage to plants.

**Figure 4:**
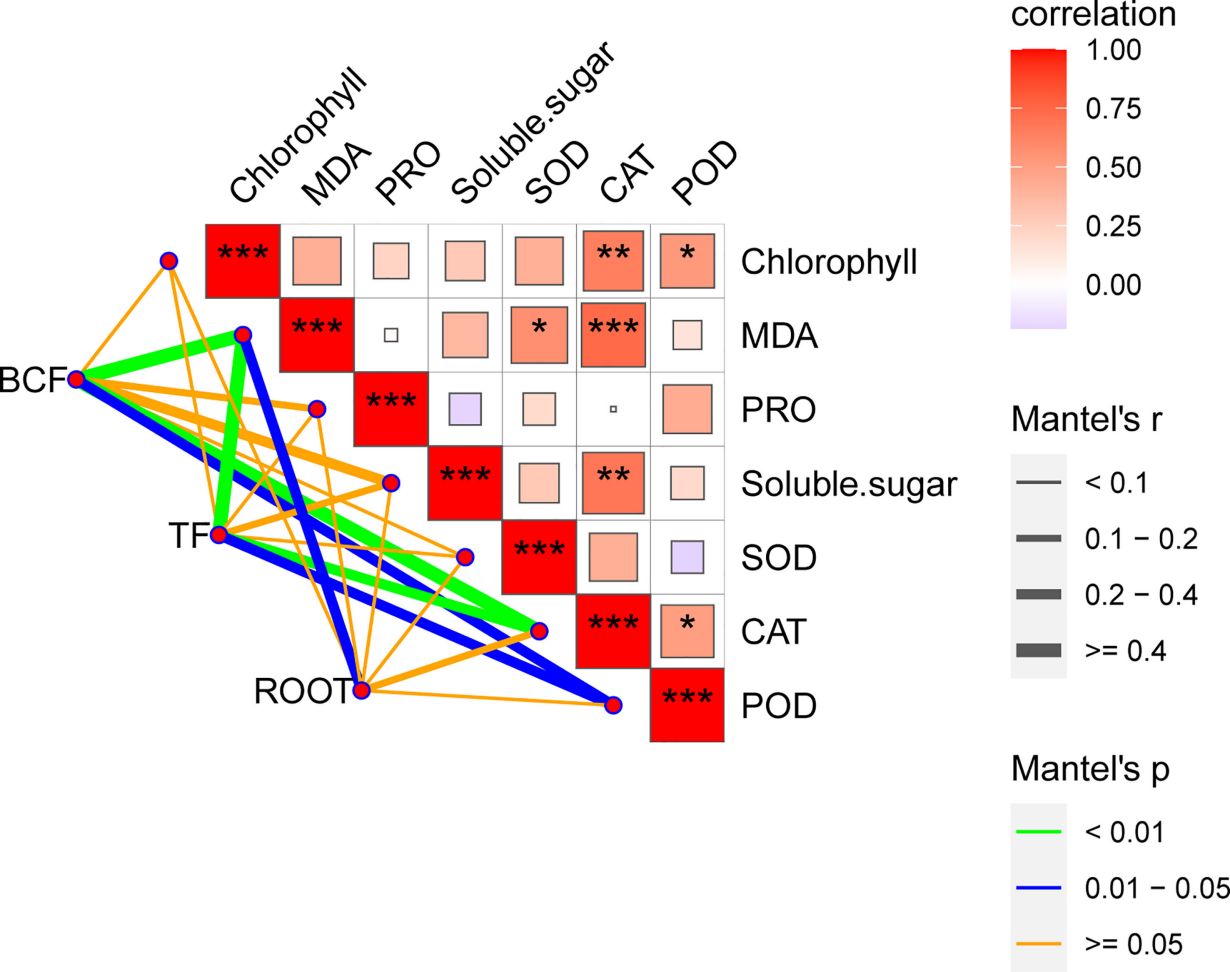
Mantel tests of BCF, TF, and root growth with physiological index variability. The color gradient represents the Spearman correlation coefficient under pairwise comparisons of physiological index variability. Edge width corresponds to Mantel’s *r* statistic of distance correlation, and edge color indicates statistical significance. *** indicated P<0.001, ** indicated P<0.01, * indicated P<0.05.

**Figure 5:**
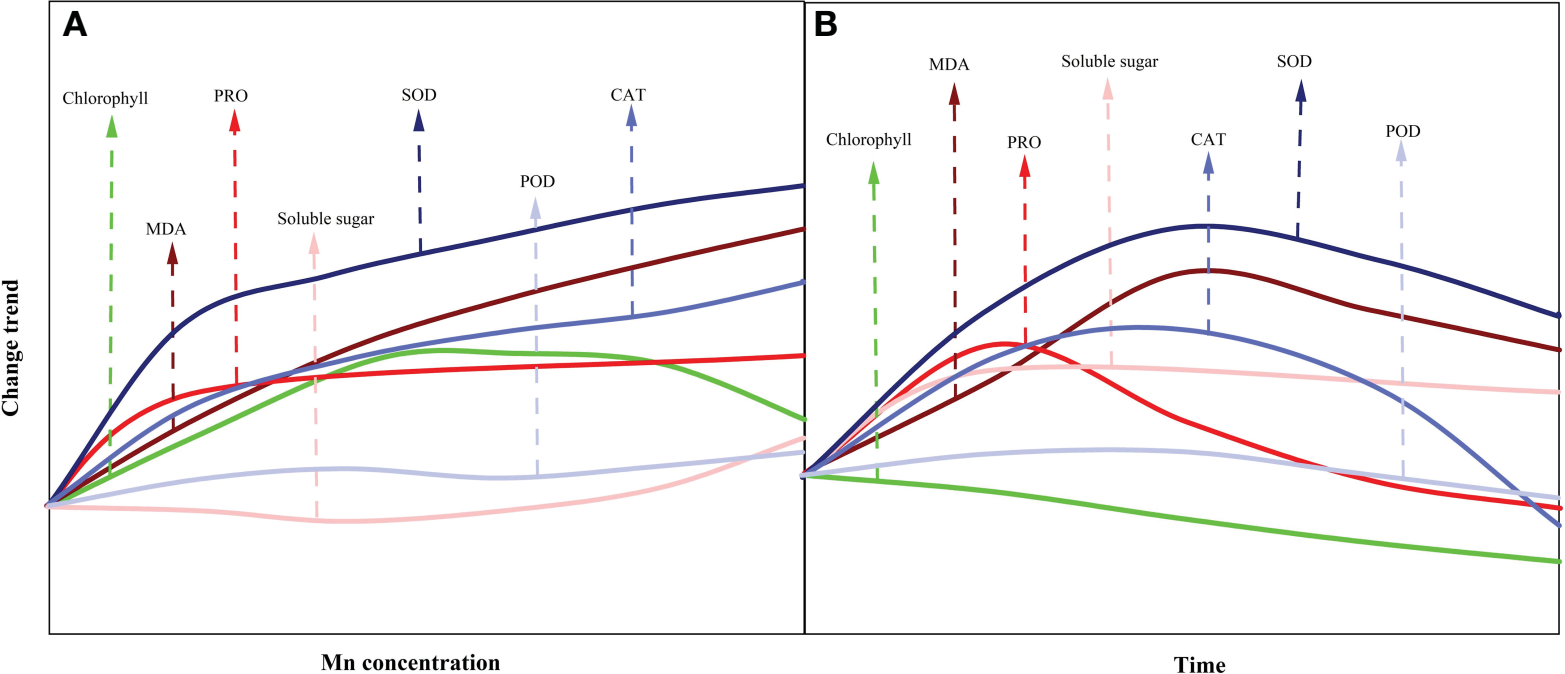
Change trend of physiological indexes with Mn concentration **(A)** and time **(B)**.

Measuring physiological responses is important as it is the link between micromolecules and macrophenotypes (Liu et al., 2023). Currently, measurement of the plant physiological indexes under adverse conditions is necessary to understand the physiological responses of plants to adversity. Our statistical analysis showed that the adsorption capacity, transfer capacity, and root growth characteristics of *B. papyrifera* to Mn were highly correlated with the limited physiological indexes (**Figure 4**, **Figure S5**). Currently, a majority of studies have only selected limited time points to measure physiological indexes and ignore the variations that may occur over time. This shortcoming in experimental design has prevented more accurate conclusions, especially for Mn, which has a typical binary effect. Importantly, physiological indexes are easily monitored and are widely used in agricultural production and forestry (Karaman, 2020). Thus, it is necessary to conduct continuous monitoring of physiological indexes for different plants under heavy metal stress and establish a database. By comparing the trends of different physiological indexes with Mn concentration and time in this study, MDA was confirmed to be a more suitable physiological indicator for monitoring the toxicity of Mn stress (**Figures 4**, **5**). At the same time, SOD and CAT are supposedly the main antioxidant enzymes for the response of *B. papyrifera* to Mn stress.

## 5 Conclusion

We used *B. papyrifera* as a potential plant for mitigating Mn pollution and noticed that this plant absorbed Mn and transferred it to the aerial parts of plants, especially the leaves. Mn also promoted the root growth of *B. papyrifera*. For the first time, the dynamic characteristics of physiological indexes of *B. papyrifera* under Mn stress were explored and Mn showed a binary effect. Mn concentration and stress time had significant effects on all measured physiological indexes of *B. papyrifera*. Except for soluble sugar, Mn concentration and stress time had significant interactions with the physiological indexes. The accumulation of Mn caused lipid peroxidation in the cell membrane, which increased the toxic substances in *B. papyrifera*, resulting in an increase in MDA, which was confirmed to be a suitable physiological indicator for monitoring toxicity. *Broussonetia papyrifera* improved the activity of antioxidant enzymes (SOD, CAT, and POD) to remove active oxygen free radicals from the plant, and SOD and CAT are supposed to be the main antioxidant enzymes for the response. Thus, in the monitoring process of Mn stress, it is necessary to select appropriate plant organs for sampling according to Mn concentration.

## Data availability statement

The original contributions presented in the study are included in the article/**Supplementary Material**. Further inquiries can be directed to the corresponding author.

## Author contributions

All authors contributed to the study conception and design. The first draft of the manuscript was written by FL, HH and XZ. FL, HH and ZY were responsible for the conceptualization. FL, HH, YG and ZM were responsible for the methodology. XZ, HH and ZY were responsible for the funding acquisition. All authors contributed to the article and approved the submitted version.

## Supplementary material

Supplementary Figure 1 The images of root structure of *B. papyrifera* under different Mn concentrations.

Supplementary Figure 2 The effects of different Mn treatment concentrations on chlorophyll content **(A)** and the effects of time on chlorophyll content **(B)** for *B. papyrifera*. Results of chlorophyll content were expressed as mean ± SD.

Supplementary Figure 3 The effects of different Mn treatment concentrations and time MDA (**A**1, 2), proline (**B**1, 2), soluble sugar (**C**1, 2). Results of content were expressed as mean ± SD.

Supplementary Figure 4 The effects of different Mn treatment concentrations and time SOD (**A**1, 2), CAT (**B**1, 2), POD (**C**1, 2). Results of content were expressed as mean ± SD.

Supplementary Figure 5 Mantel tests of BCF, TF and root growth with physiological indexes. The color gradient represents the Spearman correlation coefficient under pairwise comparisons of physiological indexes. Edge width corresponds to Mantel’s r statistic of distance correlation, and edge color indicates statistical significance.
