# Supplementary material for: Physiological dynamics as indicators of plant response to manganese binary effect
Source: Front Plant Sci. 2023 Apr 12;14:1145427. doi: 10.3389/fpls.2023.1145427 (PMC10130396; doi:10.3389/fpls.2023.1145427)
Supplement: Supplementary Figure 1 — The images of root structure of B. papyrifera under different Mn concentrations. [file DataSheet_1.docx]

Supplementary materials

Physiological dynamics as the indicators of plant response to manganese binary effect

**Xu Zhenggang^1,2^, Fan Li^2^, Zheng Mengxi^1^, Zhao Yunlin^1^, Huang Huimin^1,3^, Yang Guiyan^1,*^**

^1^ College of Forestry, Northwest A &F University, Yangling 712100, Shaanxi, China

^2^ Hunan Research Center of Engineering Technology for Utilization of Environmental and Resources Plant, Central South University of Forestry and Technology, Changsha 410004, Hunan, China;

^3^ Changsha Environmental Protection College, Changsha 410004, Hunan, China;

* Corresponding author: Yang Guiyan, yangguiyan@nwsuaf.edu.cn

| **Mn concentration** | **Sample 1** | **Sample 2** | **Sample 3** |
| --- | --- | --- | --- |
| 0 mmol/L | 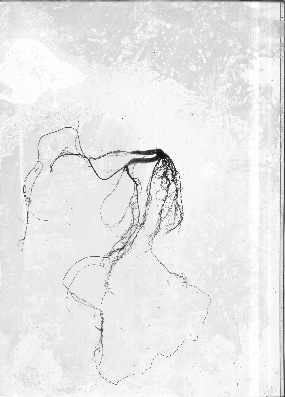 |  |  |
| 0.25 mmol/L |  |  |  |
| 0.5 mmol/L |  |  |  |
| 1 mmol/L |  |  | 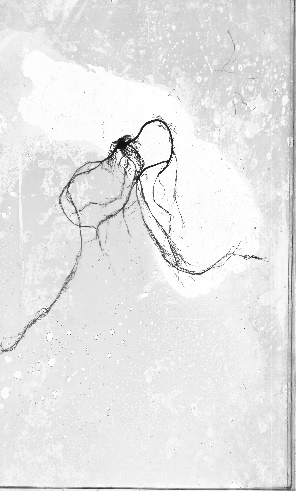 |
| 2 mmol/L | 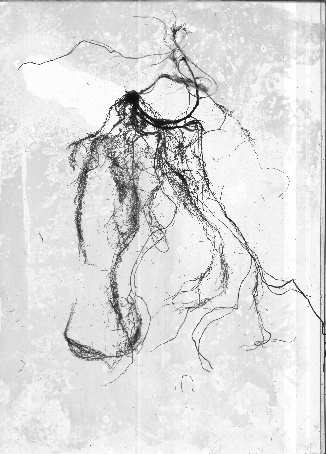 |  |  |
| 5 mmol/L | 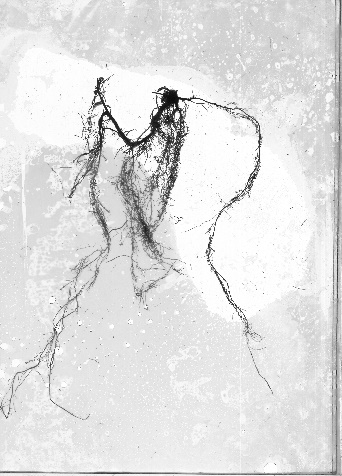 | 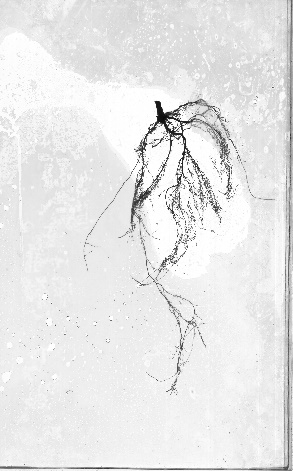 |  |

**Figure S1** The images of root structure of *B. papyrifera* under different Mn concentrations.


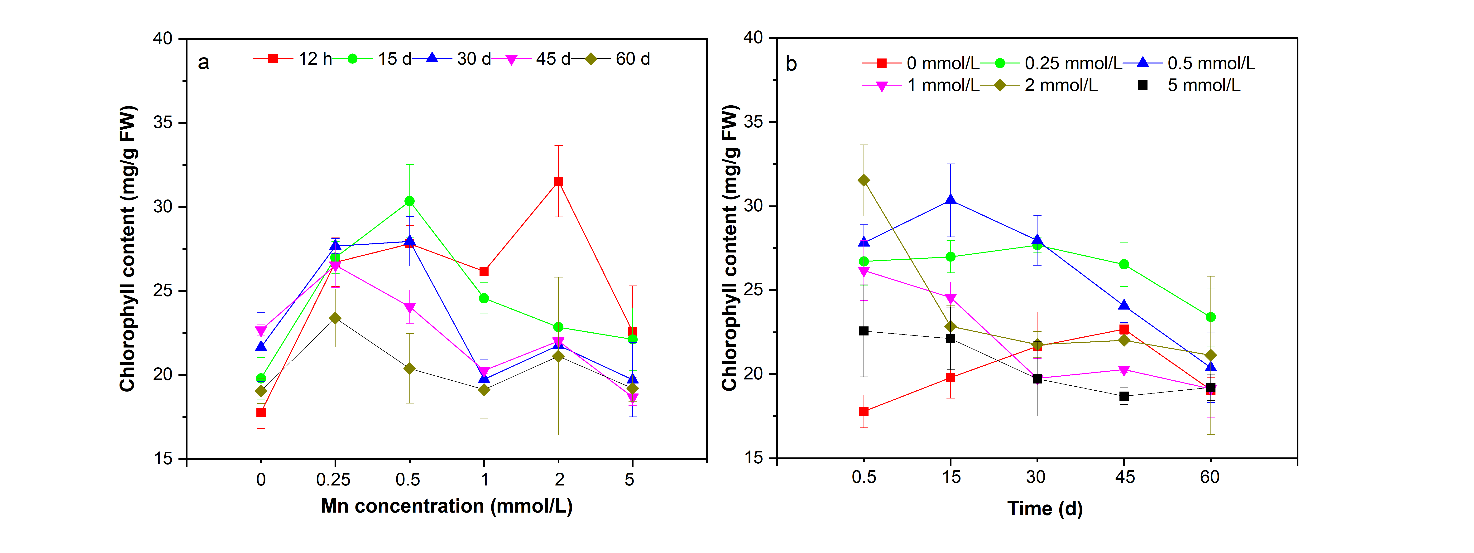


**Figure S2** The effects of different Mn treatment concentrations on chlorophyll content (a) and the effects of time on chlorophyll content (b) for *B. papyrifera*. Results of chlorophyll content were expressed as mean ± SD.


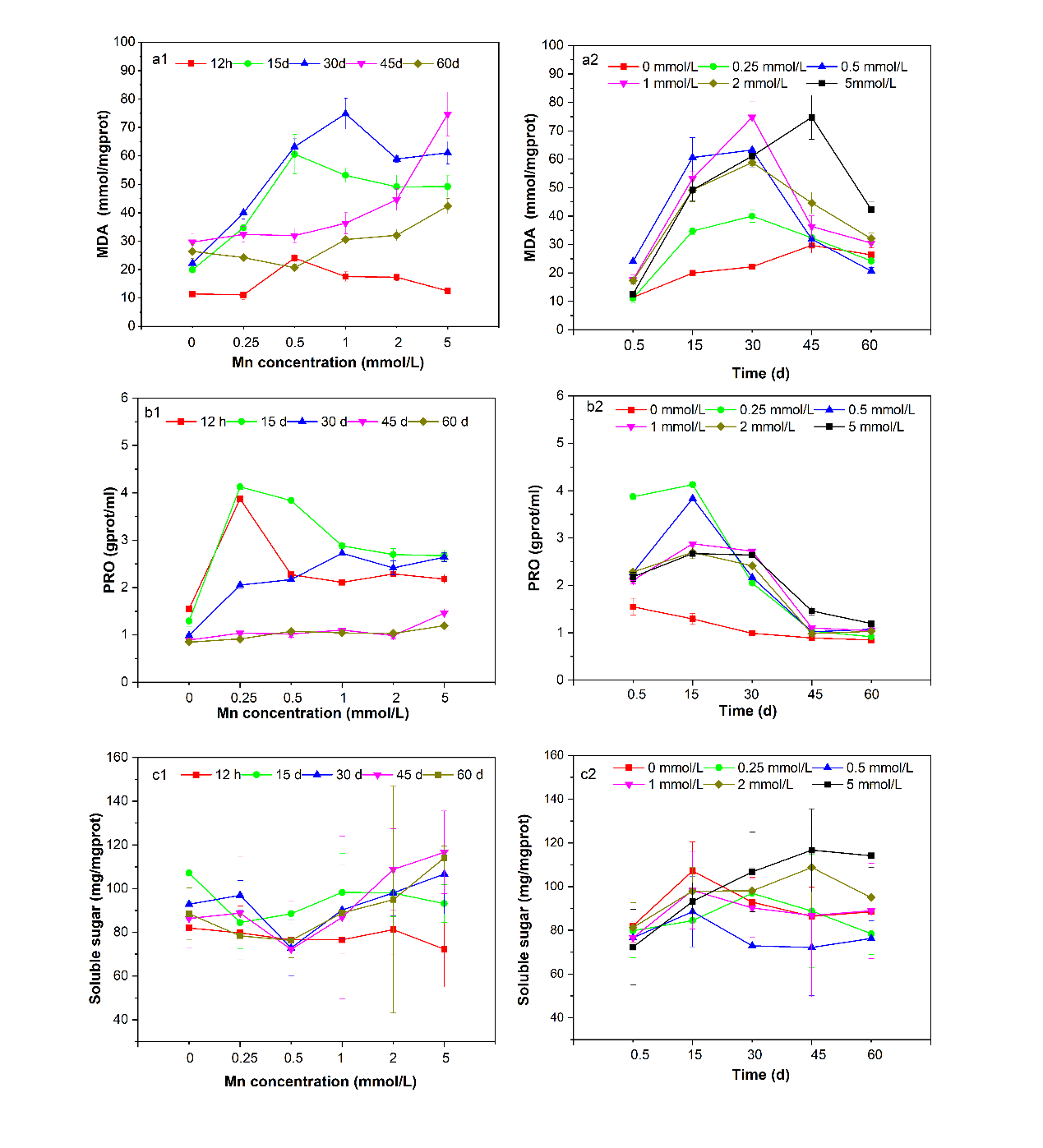


**Figure S3** The effects of different Mn treatment concentrations and time MDA (a1, a2), proline (b1, b2), soluble sugar (c1, c2). Results of content were expressed as mean ± SD.


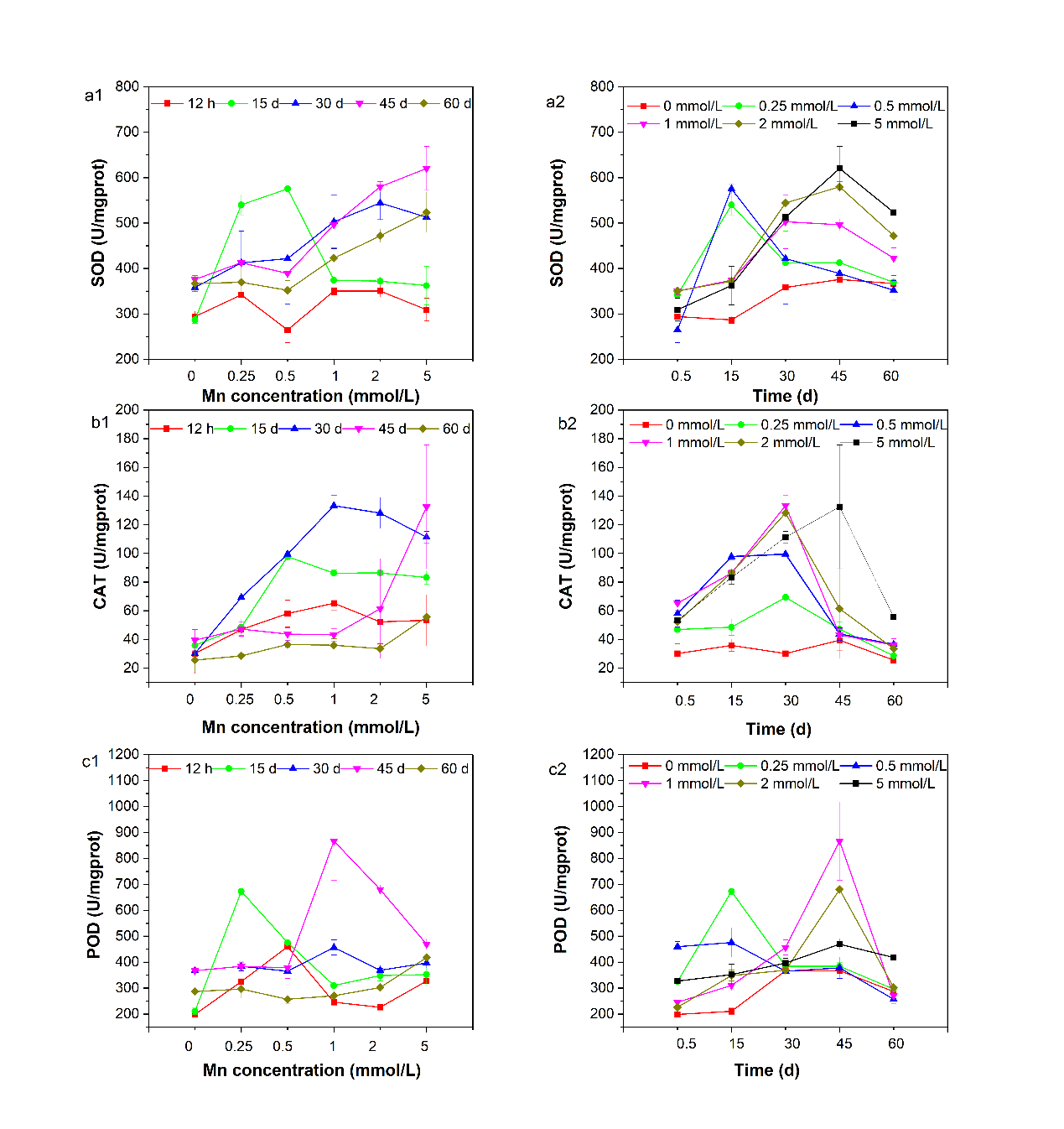


**Figure S4** The effects of different Mn treatment concentrations and time SOD (a1, a2), CAT (b1, b2), POD (c1, c2). Results of content were expressed as mean ± SD.


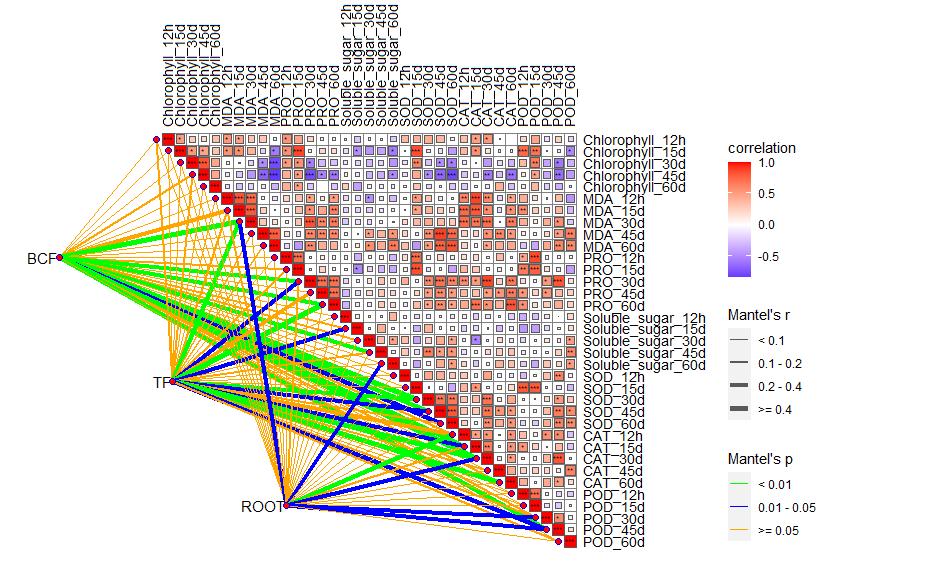


**Figure S5** Mantel tests of BCF, TF and root growth with physiological indexes. The color gradient represents the Spearman correlation coefficient under pairwise comparisons of physiological indexes. Edge width corresponds to Mantel's r statistic of distance correlation, and edge color indicates statistical significance.
